# Supplementary material for: Association of chromosomal aberrations in chromosomes 3 and 7, and P16 mutations with malignancy in salivary gland tumors
Source: PeerJ. 2025 Mar 31;13:e19217. doi: 10.7717/peerj.19217 (PMC11967423; doi:10.7717/peerj.19217)
Supplement: Supplemental Information 1 [file peerj-13-19217-s001.docx]

| Serial Number | Primer Name | Sequence |
| --- | --- | --- |
| SEQID NO:1 | CSP 3-F | GTGGAATTTGCAAGTGGAGA |
| SEQID NO:2 | CSP 3-R | CTTTTCCACCAATGGCCTCA |
| SEQID NO:3 | CSP 7-F | TGCAAGTGGAGATTTCAAGC |
| SEQID NO:4 | CSP 7-R | CAAATATCCACTTGCAGACA |

**Table S1** Sequences and Target Regions of CSP3 and CSP7 Probe Primers

| BAC Identifier | Insert Fragment Start-End Position | Size (kb) |
| --- | --- | --- |
| RP11-615P15 | chr9:21,764,403-21,981,385 | 217 |
| RP11-478M20 | chr9:21,947,304-22,110,179 | 163 |

**Table S2** BAC Clones Selected for Targeting P16


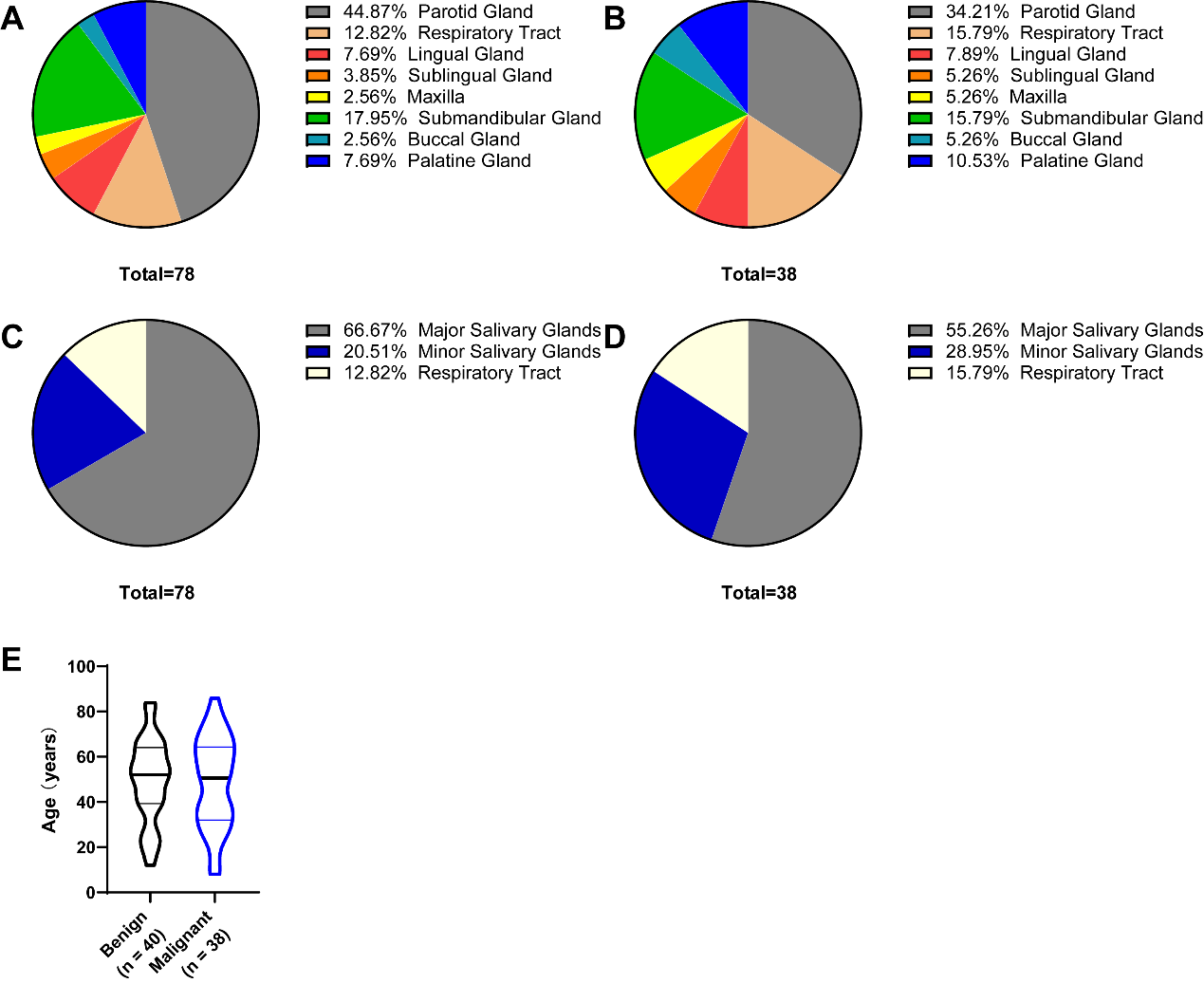


**Figure S1 Clinical data from patients were analyzed.** The tumor locations of 78 salivary gland tumor patients (A) and 38 of those with malignant salivary gland tumors (B) were examined. After classifying the types of salivary glands, the tumor locations of all 78 salivary gland tumor patients (A) and the 38 with malignant salivary gland tumors (B) were further analyzed. (E) The age distribution of patients with both benign and malignant salivary gland tumors was assessed, and the mean and interquartile range are presented.
